# Different Evolutionary Paths to Complexity for Small and Large Populations of Digital Organisms

**DOI:** 10.1371/journal.pcbi.1005066

**Published:** 2016-12-06

**Authors:** Thomas LaBar, Christoph Adami

**Affiliations:** 1 Department of Microbiology and Molecular Genetics, Michigan State University, East Lansing, Michigan, United States of America; 2 Ecology, Evolutionary Biology, and Behavior Program, East Lansing, Michigan, United States of America; 3 BEACON Center for the Study of Evolution in Action, Michigan State University, East Lansing, Michigan, United States of America; 4 Department of Physics and Astronomy, Michigan State University, East Lansing, Michigan, United States of America; University of Illinois at Urbana-Champaign, UNITED STATES

## Abstract

A major aim of evolutionary biology is to explain the respective roles of adaptive versus non-adaptive changes in the evolution of complexity. While selection is certainly responsible for the spread and maintenance of complex phenotypes, this does not automatically imply that strong selection enhances the chance for the emergence of novel traits, that is, the origination of complexity. Population size is one parameter that alters the relative importance of adaptive and non-adaptive processes: as population size decreases, selection weakens and genetic drift grows in importance. Because of this relationship, many theories invoke a role for population size in the evolution of complexity. Such theories are difficult to test empirically because of the time required for the evolution of complexity in biological populations. Here, we used digital experimental evolution to test whether large or small asexual populations tend to evolve greater complexity. We find that both small and large—but not intermediate-sized—populations are favored to evolve larger genomes, which provides the opportunity for subsequent increases in phenotypic complexity. However, small and large populations followed different evolutionary paths towards these novel traits. Small populations evolved larger genomes by fixing slightly deleterious insertions, while large populations fixed rare beneficial insertions that increased genome size. These results demonstrate that genetic drift can lead to the evolution of complexity in small populations and that purifying selection is not powerful enough to prevent the evolution of complexity in large populations.

## Author Summary

Since the early days of theoretical population genetics. scientists have debated the role of population size in shaping evolutionary dynamics. Do large populations possess an evolutionary advantage towards complexity due to the strength of natural selection in these populations? Or do small populations have the advantage, as genetic drift allows small populations to cross fitness valleys that large populations are unlikely to traverse? There are many theories that predict whether large or small populations–those with strong selection or those with strong drift–should evolve the greatest complexity. Here, we use digital experimental evolution to examine the interplay between population size and the evolution of complexity. We found that genetic drift could lead to increased genome size and phenotypic complexity in very small populations. However, large populations also evolved large genomes and phenotypic complexity. Small populations evolved larger genomes through the fixation of slightly deleterious insertions, while large populations used rare beneficial insertions. Our results suggest that both strong drift and strong selection can allow populations to evolve similar complexity, but through different evolutionary trajectories.

## Introduction

The relative importance of adaptive (i.e., selection) versus non-adaptive (i.e., drift) mechanisms in shaping the evolution of complexity is still a matter of contention among evolutionary biologists [1–6]. In molecular evolution, the role of non-adaptive evolutionary processes such as genetic drift and genetic draft are well-established [7–9]. Theoretical population-genetic principles argue that neutral evolution, not natural selection, drove the evolution of large, primarily non-functional, genomes [10–12]. Meanwhile, there exists abundant experimental evidence that natural selection is the main cause of evolutionary change [13–15], including the spread of novel adaptive phenotypes [16, 17] in experimental populations. However, it is still possible that non-adaptive processes play a significant role in the evolution of complexity. For instance, genetic drift (or relaxed selection) may allow for the accumulation of mutations that can later lead to the evolution of novel complexity [4, 18]. Much of the work demonstrating the role of selection in driving the evolution of novel complex traits is based on experiments with large populations and strong selection [19]. In much smaller populations (i.e., those with fewer than 10^4^ individuals), selection is weaker, and genetic drift begins to alter evolutionary dynamics [15, 20]. Therefore, to explain the role of adaptive vs. non-adaptive process in the evolution of complexity, one must explore the role of population size in the evolution of complexity.

Both theoretical modeling and experiments suggest many possibilities for the relationship between population size and the evolution of complexity. There are two classes of evolutionary trajectories that would favor large populations in the evolution of complexity. First, populations could perform an adaptive walk (the fixation of a sequence of beneficial mutations) towards the evolution of a novel complex trait [21]. If this was the case, then larger populations would follow this trajectory faster than small populations due to their larger mutation supply. Experiments with microorganisms support the possible existence of adaptive trajectories towards complexity, as there is strong evidence that the mutations leading up to a phenotypic innovation in both *Escherichia coli* [22] and phage *λ* [23] were under positive selection. However, it is unclear whether adaptive mutations generally precede the evolution of complex traits or whether these large microbial populations can only take adaptive walks due to the intensity of selection in large populations. The second type of trajectory that favors large populations is the neutral walk (the fixation of a sequence of neutral mutations). While any individual neutral mutation has a low probability of fixation, a large population would be able to accumulate many neutral mutations at any given time allowing for the exploration of its fitness landscape. Work by Wagner and colleagues suggests that many phenotypic traits are connected to each other by sequences of phenotypically neutral mutations [18, 24].

If the evolution of complexity requires the fixation of deleterious mutations (for example, via valley-crossing), then the elimination of deleterious mutations by purifying selection may limit the evolutionary advantage large populations may have. Wright was the first to propose an evolutionary advantage of small populations due to valley-crossing [25]. More recently, scientists have explored under which conditions small populations have an evolutionary advantage over large populations [26, 27]. A prominent theory that predicts that small (but not large) populations should evolve the greatest genomic complexity (and subsequently organismal complexity) is the Mutational Burden (or Mutational Hazard) hypothesis, proposed by Lynch and colleagues [4, 28, 29]. This hypothesis argues that genome size should be inversely correlated with the product of the effective population size and the mutation rate [3, 28]. Strong purifying selection against excessive genome size streamlines the genomes in large populations [30–32]. Meanwhile, weakened purifying selection and increased genetic drift in small populations results in the accumulation of slightly deleterious excess genome content [3, 29]. At a later time, this slightly deleterious genome content may be mutated into novel beneficial traits [4, 33]. However, recent work on valley-crossing in asexual populations (and sexual populations with a low recombination rate) showed that both small and large populations cross valleys more than intermediate-sized populations [34–36]. Therefore, it is not clear whether large or small populations are expected to evolve the greatest complexity when deleterious mutations are required.

The long timescales required to observe the emergence of novelty and evolution of complexity make biological experiments to distinguish between these theories difficult to perform. To overcome this difficulty, we used digital experimental evolution [37] to test the role of population size on the evolution of genome size and phenotypic complexity in asexual organisms. Digital evolution has a long history of addressing macroevolutionary questions (such as the evolution of novel traits) experimentally [38, 39]. Digital populations can be manipulated in ways that biochemical organisms can not, making it possible to study aspects of the evolutionary process that are ordinarily too difficult to test [40]. In this regard, digital experimental evolution has the same goals as microbial experimental evolution: to use a well-controlled model system that is as simple as possible, to study “evolution in action” [41]. And while digital evolution studies cannot test hypotheses that depend on particular biochemical processes involved in cellular life, digital populations do undergo selection, drift, and mutation, allowing for their use in testing hypotheses derived from theoretical population genetics. Thus, digital experimental evolution represents a well-suited model system to test the population genetics-based theories concerning the role of population size in the evolution of complexity.

Here, we evolved populations ranging in size from 10 to 10^4^ individuals, starting with a minimal-genome ancestor. We found that small populations do evolve greater genome sizes and phenotypic complexity (number of phenotypic traits) than intermediate-sized populations. These small populations evolve larger genomes primarily through increased fixation of slightly deleterious insertions. However, the small population sizes that enhance the evolution of phenotypic complexity also enhance the likelihood of population extinction. We also found that the largest populations evolved similar complexity to the smallest populations. Large populations evolved longer genomes and greater phenotypic complexity through the fixation of rare beneficial insertions instead. Large populations were able to discover these rare beneficial mutations due to an increased mutation supply. Finally, we found that a strong deletion bias can prevent the evolution of greater complexity in small, but not in large, populations.

## Results

To explore the effect of population size on the evolution of genome size and phenotypic complexity, we used the Avida digital evolution system [42]. Avida is a platform that allows researchers to perform evolution experiments inside of a computer, as the genetic code that evolves are actual computer programs of variable length. It has been used extensively in research in evolutionary biology [37, 43, 44], and is described in detail in Methods.

We evolved one hundred replicate populations across a range of population sizes (10 − 10^4^ individuals) for 2.5 × 10^5^ generations. Many of the smallest populations (those with ten individuals) did not survive the entire experiment. Therefore, we evolved one hundred additional small populations ranging from twenty individuals to ninety individuals in order to examine how the probability of extinction was related to the evolution of complexity. All populations with at least thirty individuals survived for the entire experiment. Forty-seven of the populations with ten individuals went extinct, while only one of one hundred populations underwent extinction in the populations with twenty individuals. Extinction was a consequence of populations evolving large genomes that accumulated deleterious mutations and led to the production of only non-viable offspring. These extinct populations were not included in the statistics described below.

### Genome Size Evolution

**Fig 1:**
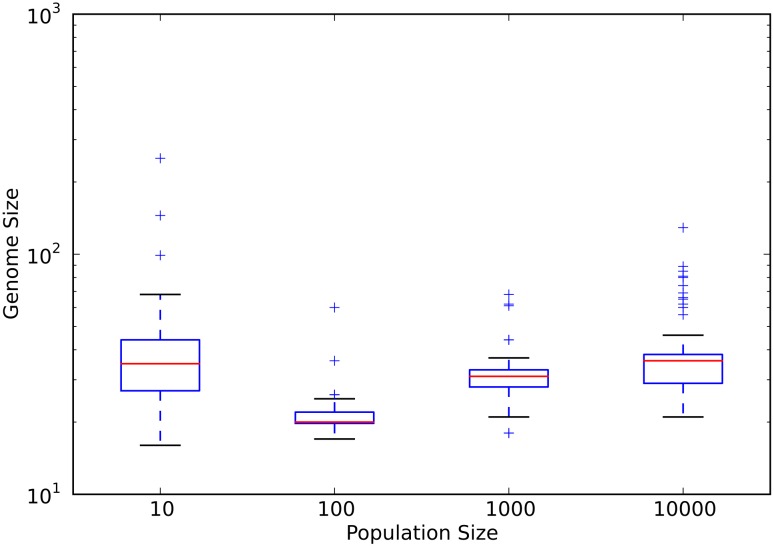
Final genome size as a function of population size. Red lines are the median values for each population size. The upper and lower limits of each box denote the third and first quartile, respectively. Whiskers are 1.5 times the relevant quartile value. Plus signs denote those data points beyond the whiskers. Data represent only those populations that did not go extinct.

Of the surviving populations, we first examined how genome size changes from the ancestral value of fifteen instructions. The size of the genome from every population size increased, on average (see Fig 1 and panel A in S1 Fig). However, both the smallest and the largest populations evolved the largest genomes. Populations with ten individuals evolved a median genome size of 35 instructions, while populations with ten thousand individuals evolved a median genome size of 36 instructions. The median final genome size decreased as population size increased for populations with between ten and fifty individuals. However, from populations with fifty individuals to populations with ten thousand individuals, the median final genome size increased as population size increased.

**Fig 2:**
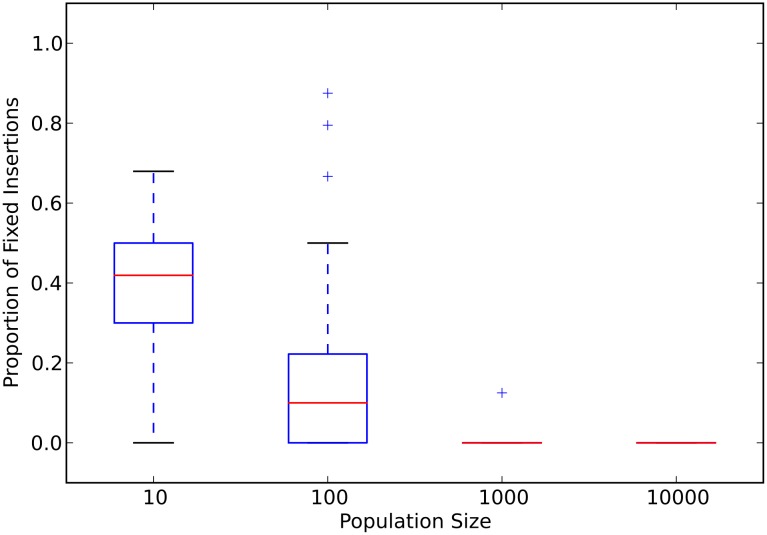
Proportion of slightly-deleterious insertions as a function of population size. Red lines are the median values for each population size. The upper and lower limits of each box denote the third and first quartile, respectively. Whiskers are 1.5 times the relevant quartile value. Plus signs denote those data points beyond the whiskers. Data represent only those populations that did not go extinct.

**Fig 3:**
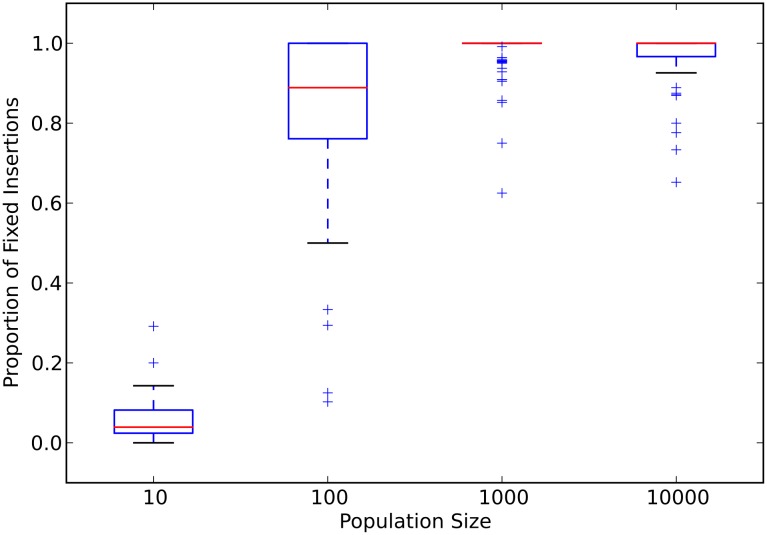
Proportion of insertions under positive selection as a function of population size. Red lines are the median values for each population size. The upper and lower limits of each box denote the third and first quartile, respectively. Whiskers are 1.5 times the relevant quartile value. Plus signs denote those data points beyond the whiskers. Data represent only those populations that did not go extinct.

Next, we examined the dynamics of fixation of insertion mutations (insertions, for short) to explain why both the smallest and the largest populations evolved the largest genomes. For each experimental population, we counted every insertion that occurred on the fittest genotype’s ancestral lineage that went back to the ancestral genotype (the “line of descent”, see Methods). The median number of insertions fixed follows the same trend as the evolution of genome size (S2 Fig). A large fraction of these fixed insertions are slightly deleterious in populations with fewer than one hundred individuals (see Fig 2 and panel B in S1 Fig). However, no insertions are slightly deleterious, on average, in large populations with more than one hundred individuals. The opposite trend holds for beneficial insertions. The fraction of insertions that are under positive selection increases with increasing population size, with the largest populations usually fixing only beneficial insertions (Fig 3 and panel C in S1 Fig). These data demonstrate that small populations evolve larger genomes through the fixation of slightly deleterious insertions. However, large populations can evolve similarly large genomes through the fixation of rare beneficial insertions.

### Evolution of Phenotypic Complexity

**Fig 4:**
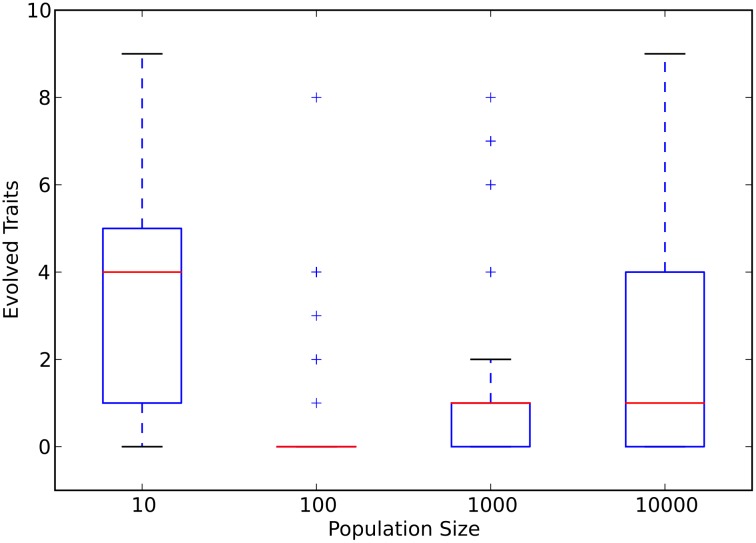
Final number of evolved phenotypic traits as a function of population size. Red lines are the median values for each population size. The upper and lower limits of each box denote the third and first quartile, respectively. Whiskers are 1.5 times the relevant quartile value. Plus signs denote those data points beyond the whiskers. Data represent only those populations that did not go extinct.

Next, we focus on the role of population size in the evolution of phenotypic complexity (defined as the number of phenotypic traits). In Avida, a phenotypic trait is a program’s ability to perform a certain mathematical operation on binary numbers (see Methods). The evolution of phenotypic complexity follows the same trend as the evolution of genome size (see Fig 4 and panel D in S1 Fig). Populations with ten individuals evolved a median of four traits, while populations with one thousand and ten thousand individuals evolved a median of one trait. The rest of the population sizes evolved a median of zero traits. As an avidian’s fitness is primarily determined by its phenotypic traits in the Avida environment used here, the evolution of fitness showed a similar trend to the evolution of phenotypic complexity (S3 Fig).

**Fig 5:**
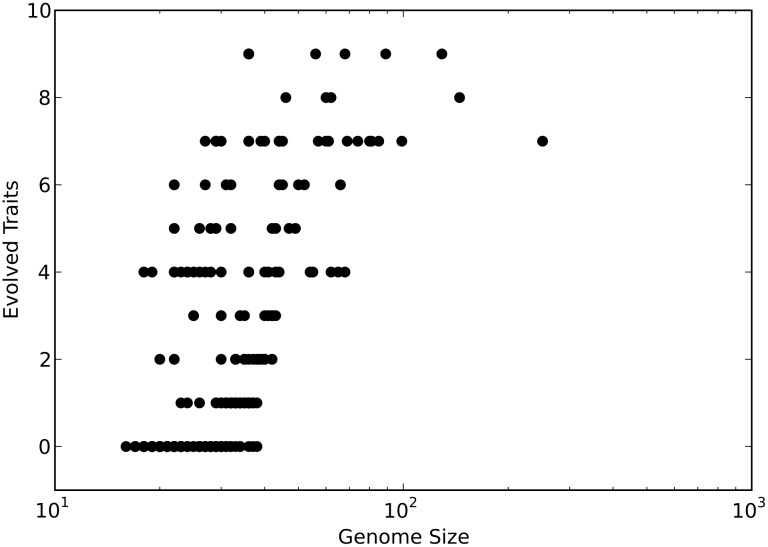
Correlation between the final genome size and the final number of evolved traits. Black circles represent the combined data from populations with 10, 100, 1000, and 10000 individuals. Only replicates that survived all 2.5×10^5^ generations were included.

That the trend in genome size evolution and in phenotypic complexity evolution are mirrored suggests that the evolution of larger genomes enables the evolution of increased phenotypic complexity. To establish a link between the two, we performed two tests. First, we examined the correlation between genome size and phenotypic complexity across all populations. Phenotypic complexity is positively correlated with genome size (Fig 5, Spearman’s *ρ* ≈0.72; *p* < 2.3 x 10^−57^), suggesting that it was the increased genome size that allowed for the evolution of increased phenotypic complexity. However, there are two potential mechanisms that could cause an increased genome size to result in increased phenotypic complexity. On the one hand an increased genome size could simply allow more “room” for novel functional content. On the other hand, it could be that the increased genome size leads to a faster rate of evolution due to the increased genomic mutation rate. To examine the role of an increased mutation rate in driving the evolution of phenotypic complexity, we evolved a further one hundred populations of ten individuals with a fixed genomic mutation rate of 1.5 × 10^−1^ (i.e., the ancestral genomic mutation rate). Under this condition, no population went extinct (as opposed to forty-seven in the variable mutation rate treatment). The fixed genomic mutation rate populations evolved a median of 2 phenotypic traits compared to the variable genomic mutation rate populations that had evolved a median of 4 phenotypic traits (S4 Fig). These data demonstrate that the increased genomic mutation rate that follows from larger genomes does increase the evolution of phenotypic complexity. However, even with a fixed genomic mutation rate, the smallest populations still evolved a greater median number of traits (on average 2 traits) than every other population size. Thus, while an increased genomic mutation rate (due to increased sequence length) indeed enhances the evolution of phenotypic complexity, small populations still possess an evolutionary advantage due to drift-driven increases in genome size only.

### Non-functional Insertions

In the previous experiments, large populations evolved larger genomes and greater phenotypic complexity because they fixed rare beneficial insertions. Next, we more closely examine the finding that beneficial insertions are necessary for the evolution of complexity in large populations. We repeated the experiments with the same population sizes and mutation rates, except we changed how insertions worked. Instead of inserting one of the twenty-six instructions that compose the Avida instruction set, we inserted “blank” instructions into the genome (see Methods for details). These blank instructions cannot be beneficial (on their own or in combination with existing instructions) and would have to be further mutated to lead to the evolution of phenotypic complexity. In this treatment, greater phenotypic complexity in large populations would require a two-step mutational process, as opposed to the single step in a beneficial insertion.

We saw no qualitative difference in the trend between these experiments and the original experiments (S5 Fig). Very small and large populations still both evolved the largest genomes and the greatest phenotypic complexity. Populations of all sizes evolved longer genomes and more phenotypic traits in this treatment (S5 Fig) than in the original treatment (Figs 1 and 4). The fraction of fixed insertions that were under positive selection decreased for every population size compared to the original experiments, as expected from the insertion of non-functional instructions (S6 Fig). We observed an increased rate of extinction in the very small populations, with only 2 populations with ten individuals and 25 populations with twenty individuals surviving the experiment. Population extinction was likely enhanced by the increased growth in genome size in these experiments as compared to the original experiments.

### Deletion Bias

Finally, we performed experiments to test whether the effect of a deletion bias (a higher fraction of deletions among all indels) alters the relationship between population size and the evolution of complexity. A biased ratio of deletion to insertion mutations is found in biological organisms across the tree of life, especially in bacteria [45, 46]. In these experiments we set the ratio of deletions to insertions as 9:1, but kept the total indel mutation rate as in the original experiments. In this treatment, only one population with ten individuals went extinct, as opposed to 47 populations in the original treatment. However, the advantage towards evolving complexity previously enjoyed by small populations vanished (S7 Fig). The median genome size increased as the population size increased for all populations sizes. Only the largest populations evolved a median number of novel phenotypic traits greater than zero. These results suggest that it is not only the role of genetic drift, but the equal frequency of insertions and deletions that results in the increased genome size and phenotypic complexity in small populations.

## Discussion

The idea that small populations could have an evolutionary advantage over large populations dates back to Wright and his Shifting Balance theory [25]. More recently, a potential small-population advantage has been demonstrated both theoretically [27] and experimentally [26], but only in regard to short-term increases in fitness. The Mutational Burden hypothesis provides an evolutionary mechanism that gives small populations an advantage towards increased phenotypic complexity [4, 33]. However, an experimental demonstration of this advantage is lacking. Our study provides further insight into the conditions that give small populations such an evolutionary advantage. We confirmed that small populations do evolve larger genomes due to the increased fixation of slightly deleterious mutations, as predicted [28]. We also showed how small populations have an increased potential to later evolve increased phenotypic complexity in small populations through the larger genomes generated by increased genetic drift [3, 4]. As phenotypic traits are strongly beneficial in the Avida environment used here, these small populations used slightly deleterious genome expansions to cross fitness valleys and eventually reach novel fitness peaks.

Our work also shows that this evolutionary advantage of small populations is limited by an increased rate of population extinction. Such a trend between the evolution of large genomes and an increased rate of extinction is seen in some multicellular eukaryote clades [47, 48]. These small populations are still likely to have a larger risk of extinction beyond that caused by population-genetic risks such as Muller’s ratchet [49] and mutational meltdowns [50, 51]. Ecological stressors increase extinction risk [52] and small populations are less able to adapt to detrimental environmental changes [53]. Our results concerning extinction, combined with the risk of other factors not examined here, suggest that the likelihood of a small population using genetic drift to evolve greater complexity without an increased risk of extinction may be limited. However, it is possible that multiple small populations could reduce the risk of extinction without reducing the evolution of complexity; future work should consider the interplay between population size and the evolution of complexity within a metapopulation of small populations.

Large populations also evolved greater genome sizes and phenotypic complexity. In our original experiments, genome evolution in large populations was driven by the fixation of rare beneficial insertions (Fig 4). While it is likely that many gene duplications are not under positive selection and lost due to genetic drift and mutation accumulation [54], some, especially those resulting in the amplification of gene expression, can be immediately beneficial and later lead to increased phenotypic complexity [55–58]. Due to the increased mutation supply, these events would occur at a greater frequency in large populations [59] and possibly lead to an increased probability of the evolution of complexity there. However, we also found that large populations did not require this large supply of beneficial insertions. Even when insertion mutations added non-functional instructions and further point mutations were required to evolve functional traits, large populations still evolved complexity similar to that evolved in small populations. These results suggest that purifying selection may not limit the evolution of complexity in large populations. Finally, we found that when deletions occur at a much greater frequency than insertions, only large populations have an evolutionary advantage towards complexity. As many bacteria do have a bias towards deletions [60, 61], this result suggests that large microbial populations can have an evolutionary advantage over small microbial populations for evolving novel traits after all.

Such a trend where both large and small, but not intermediate-sized populations have an evolutionary advantage has already been theoretically proposed elsewhere. Weissman et al. showed that both small and large populations cross fitness valleys more easily than intermediate-sized populations [34]. Small populations valley-crossed due to genetic drift and large populations did so due to an increased supply of double mutants. Ochs and Desai also showed that intermediate-sized populations evolved to a lower fitness peak compared to small or large populations when valley-crossing was required for reaching a higher peak [36]. We found similar results, but from different evolutionary mechanisms. Here, populations needed to increase in genome size in order to evolve phenotypic complexity. Additionally, our populations evolved in a complex fitness landscape with many different possible paths to phenotypic complexity. While small populations did fix deleterious insertions to increase genome size, large populations evolved on a different path, either through beneficial insertions (Fig 3) or neutral insertions (S4 Fig). It is possible that even larger populations than those evolved here would fix more deleterious insertions, as the likelihood of a further, beneficial mutation arising on the background of a segregating deleterious mutation increases as population size increases. However, our results emphasize that large populations may not be dependent on valley-crossing in some fitness landscapes if alternative evolutionary trajectories exist, even if these trajectories are rare. While the first maps of fitness landscapes suggested mutational paths are small in number [62], more recent work suggests that many indirect evolutionary trajectories exist in larger fitness landscapes [63].

The population sizes that led to the evolution of greater phenotypic complexity via drift are very small (10 individuals). As biological populations of that size are unrealistic, we may wonder whether such populations can actually evolve greater complexity due to increased genetic drift. However, there are reasons to believe that these results would generally hold for biological systems. The limited range of small population sizes that led to complexity is an Avida-specific result due to the severe fitness effect of insertion mutations in avidians with small sequence length. We found that for those sequences, most insertions are lethal (about 80%), and the rest are significantly detrimental, of the order 10% to 90%. To overcome a detrimental effect of 20% via drift, populations must be as small as *N* = 10. Insertion mutations in biological genomes are not nearly as detrimental, and therefore the critical population size to see evolution of complexity via drift is much larger. In *E. coli*, for example, the deleterious effect of insertions is between 1% and 3% [64]. We can therefore expect to see the effect of increased complexity due to drift in biological populations that are small, but not unreasonably small.

Another possible avenue for future work suggested by this study is to use a simpler population genetics model to explore the same questions we attempted to answer here. Many previous theoretical studies have examined the relevance of valley-crossing to the evolution of complex traits in simple fitness landscapes [34–36]. One benefit of a simpler model is that it allows for a broader exploration of the relevant parameters involved in the interplay between population size, genome size, and the evolution of phenotypic complexity. While we were not able to perform large parameter searches using the Avida system, our work here establishes a possible relationship between the factors that influence the evolution of complexity in a fitness landscape with many possible mutational trajectories to novel traits [65]. These results should drive future theoretical studies on the evolution of genome size and phenotypic complexity using population genetics models with simpler fitness landscapes.

Here we studied the evolution of complexity in haploid asexual digital organisms with an ancestral minimal genome on a frequency-independent fitness landscape. While beyond the scope of this work, it is worth considering how adjusting these genotype characteristics would alter our results. It is likely that the ancestral minimal genomes are a requirement for small populations to evolve the same number of novel traits as large populations. If the ancestor organism had a significant amount of non-functional genome content, the mutation supply advantage that large populations have should result in an accelerated rate of phenotypic evolution in large populations [66]. The organisms used here, as in all Avida experiments, are haploid. It is possible that polyploidy would alter the results found here. However, the implementation of a ploidy cycle in Avida is non-trivial due to the mechanistic style of replication, and so presently other experimental systems would have to be used to explore the role of ploidy in the evolution of phenotypic complexity.

It is unclear how sexual, instead of asexual, reproduction would change the results. While sexual reproduction can enhance adaptation by combining beneficial mutations that arise in different background, it can also break up beneficial combinations of mutations [67]. One result that may be altered by sexual reproduction is the rate of extinction in small populations, as sex has been found to reduce the rate of mutational meltdowns [68]. Weissman et al. also demonstrate that the large population advantage towards valley-crossing does not exist under high recombination rates [35]. Sexual reproduction has previously been studied using Avida, but it is more akin to homologous recombination in bacteria [69] (as there is no ploidy cycle). Future work should address the role of sexual recombination on the results shown here. Finally, the experiments performed here had no frequency-dependent fitness effects. Previous Avida studies showed that frequency-dependent interactions enhanced the evolution of complexity for a given population size [70, 71]. It is worth exploring how the presence of frequency-dependent selection alters the evolution of complexity, especially in small populations. The benefits of the diversity seen in frequency-dependent fitness landscapes may be reduced in small populations. The extensions to the experiments performed here would provide a more complete understanding of the role of adaptive and non-adaptive evolutionary processes in the origins of complexity.

## Methods

### Avida

In order to experimentally test the role of population size and genetic drift in the evolution of complexity, we used the digital evolution system Avida version 2.14 [42]. In Avida, self-replicating computer programs (avidians) compete in a population for a limited supply of CPU (Central Processing Unit) time needed to successfully reproduce. Each avidian consists of a circular haploid genome of computer instructions. During its lifespan, an avidian executes the instructions that compose its genome. After executing certain instructions, it begins to copy its genome. This new copy will eventually be divided off from its mother (reproduction in most Avida experiments is asexual). Because an avidian passes on its genome to its descendants, there is heredity in Avida. As an avidian copies its genome, mutations may occur, resulting in imperfect transmission of hereditary information. This error-prone replication introduces variation into Avida populations. Finally, avidians that differ in instructions (their genetic code) also likely differ in their ability to self-replicate; this results in differential fitness. Therefore, because there is differential fitness, variation, and heredity, an Avida population undergoes evolution by natural selection [72]. This allows researchers to perform experimental evolution in Avida as in microbial systems [19, 73]. Avida has been successfully used as a model system to explore many topics concerning the evolution of complexity [2, 65, 71, 74, 75].

Twenty-six different instructions compose the Avida instruction set (see [42] for a more complete overview). These include instructions for genome replication, such as an instruction to allocate memory for a new daughter genome, an instruction to copy instructions from the mother genome into the daughter genome, and an instruction to divide off the new avidian. There are instructions that allow for the input, output, and manipulation of random numbers that are used in the performance of certain Boolean logic calculations (see below). There are also instructions for altering instruction execution, including conditional instructions and instructions for changing the next instruction location in the genome to be executed. It is important to note that the Avida instruction set was not designed to mimic any biological organism. Instead, it was created in order to have an organism with mechanistic reproduction in a non-specified fitness landscape that allows for studies of evolutionary dynamics.

The Avida world consists of a toroidal grid of *N* cells, where *N* is the (maximum) population size. When an avidian successfully divides, its offspring is placed into a cell in the population. While the default setting places the offspring into one of nine neighboring cells of the parent, here the offspring is placed into any cell in the entire population. This simulates a well-mixed environment without spatial structure. When there are empty cells in the population, new offspring are preferentially placed in an empty cell. However, if the population is at its carrying capacity, the individual who is currently occupying the selected cell is replaced by the new offspring (a new individual can also eliminate its parent if that cell is selected). This adds an element of genetic drift into the population as the individual to be removed is selected without regard to fitness. A population can also decrease in size by the death of individuals. An avidian will die without producing offspring if it executes 20*L* instructions without successfully undergoing division, where *L* is the avidian’s genome size. This can lead to population extinction in very small populations.

Time in Avida is divided into updates, not generations. This method of keeping time was implemented in order to allow individuals to execute their genomes in parallel. During one update, a fixed number of instructions is executed across the entire population. The resource that is necessary to execute instructions (the CPU “energy”) is measured in SIPs (single instruction processing) units. By default, there are 30*N* SIPs available to the entire population per update, where *N* is the population size. SIPs are distributed among the individual genotypes within a population in proportion to the trait or traits displayed by an individual. The total amount of SIPs garnered by an individual from traits is called the “merit”. In a homogeneous population of one genotype (clones) where each individual has the same merit, each individual will obtain approximately 30 SIPs per update. However, in a heterogeneous population where merit differs between individuals, SIPs will be distributed in an uneven manner. That way, individuals with a greater merit will execute and/or replicate a larger proportion of their genome per update and replicate faster, thus having a greater fitness. This places a strong selection pressure on evolving a greater merit. One generation has passed when the population has produced *N* offspring. Typically (depending on the complexity of an avidian) between 5 and 10 updates pass in one generation.

A genotype’s merit is increased through the evolution of certain phenotypic traits that form a “digital metabolism” [37]. These phenotypic traits are the ability (or lack there-of) to perform certain Boolean logic calculations on random binary numbers that the environment provides. To do this, an avidian must have the right “genes”–in this case, the right sequence of instructions. First, during an avidian’s lifespan, instructions that allow for the input and output of these random binary numbers must be executed. Further instructions should manipulate those numbers so as to perform the rewarded computations. When a number is then written to the output, the Avida program checks to see whether a logic operation was successfully performed. If so, the the individual that performed the computation consumes a resource tied to the performance of that trait (there are many different codes, that is, combinations of instructions, that will trigger the reward). Resource consumption causes the offspring of that individual to have their merit modified by a factor set by the experimenter. Here, we use the “Logic-9” environment to reward the performance of nine one- and two-input logic functions [65]; see S1 Table for the names and specific rewards of each function. Each individual only gains a benefit from performing each function once per generation. There is an infinite supply of resources for the performance of each logic function in the present experiments, making fitness frequency-independent. Because the performance of these logic functions increases merit, they also increase fitness and are under strong positive selection.

While increases in an individual’s merit increase replication speed and thus the individual’s fitness, fitness in Avida is implicit and not directly calculated. Unlike simulations of evolutionary dynamics, a genotype’s fitness is thus not set *a priori* by the experimenter. The only way to measure the fitness of an avidian is to run it through its lifecycle and examine its phenotype. This is similar in principle to how bacterial fitness cannot be calculated by examining an individual bacterium’s genome, but must be measured through a number of different experiments, such as competition assays [76]. A genotype’s fitness is determined by how many offspring it can produce per unit time. Genotypes that can reproduce faster will out-compete other genotypes, all else being equal. Therefore, evolution will increase a population’s fitness through two means. The first is that the population will evolve individuals with a greater number of phenotypic traits and thus with a greater merit, as explained above. The second way to increase replication speed is by optimizing (shortening) the replication time. This occurs either by shrinking the genome, which results in fewer instructions that need to be copied and replicated, or by optimizing genome architecture for faster replication. Fitness *w* in Avida can be estimated by the following equation:

$$w\approx \frac{\text{merit}}{\text{replication}\;\text{time}}$$ (1)

For an avidian to be able to successfully reproduce, it must first allocate memory for the new individual, copy its genome into the allocated memory space, and then divide off the daughter organism. As instructions are copied, the avidian may inaccurately copy some instructions into the newly allocated memory at a rate set by the experimenter. Additionally, upon division, insertions and deletions of a single instructions occur at (possibly different) rates set by the experimenter. Finally, larger insertions or deletions (indels) can occur when an avidian divides into two daughter genomes if the division occurs unevenly. In most cases, this results in the creation of one larger and one smaller genome and both of these are non-viable. However, in rare cases, one of these new genotypes is able to reproduce, resulting in a large change in genome size in that individual’s descendants. Because this mutation through inaccurate division is a characteristic of a genome and thus emergent, the rate at which it occurs is not set by the experimenter.

### Experimental Design

We used four experimental designs (treatments) to explore how population size determines the evolution of complexity: the original experiments, the non-functional insertion experiments, the fixed genomic mutation rate experiments, and the deletion bias experiments. For all experiments, we evolved populations of size *N* = {10,100,1000,10000} for 2.5 × 10^5^ generations under 100-fold replication. For the original treatment, we also performed experiments with population sizes of *N* = {20,30,40,50,60,70,80,90}. All populations were initiated at full size *N* with an altered version of the standard length-100 Avida start organism [42]. The alteration was the removal of all non-essential genome content (85 nop-c instructions). This reduced the genome size of the ancestor organisms from 100 instructions to only 15 instructions.

For the original experiments, point mutations occurred at a rate of 0.01 mutations per instruction copied, and insertions and deletions at 0.005 events per division. Insertions and deletions occur at most once per division. The ancestor thus started with a genomic mutation rate of 0.15 mutations per generation (0.01 mutations/instruction copied × fifteen instructions copied per generation), but this changes over the course of the experiment as genome size evolves. These experiments are similar to most standard Avida experiments, with the exception of a smaller genome size (fifteen instructions) for the ancestral organism.

For the remainder of the experimental settings, one of the above settings was changed to examine a specific effect. For the experiments where the genomic mutation rate was fixed, point mutations occurred at a rate of 0.15 mutations *per division*, independently of genome size, which fixes the mutation rate at 0.15 mutations/genome/generation. For the non-functional insertion experiments, the mutation rates were the same as in the original experiments. However, instead of inserting one of the twenty-six instructions from the Avida instruction set (see [42] for the Avida instruction set), “blank” instructions called nop-x were inserted. These instructions have no function on their own or in combination with any other instruction. Finally, for the deletion bias experiments, point mutations occurred at the same rate as in the standard experiments. However, insertions and deletions did not occur at the same rate. Insertions occurred at a rate of 0.001 per division and deletions occurred at a rate of 0.009 per division. This kept the total mutation rate equal to the other experimental treatments, while altering the ratio of insertions to deletions.

### Data Analysis

In order to analyze the evolution of complexity in each population, we extracted the individual with the greatest fitness at the end of each experiment (the “dominant” type). We then calculated relevant statistics for each of these genotypes by running them through Avida’s *analyze mode*. This mode allows us to run each genotype through its lifecycle in isolation, and calculate its fitness, its genome size, whether it performs any logic functions, and whether it produces viable offspring, among other characteristics. To measure the evolution of phenotypic complexity, we determined how many unique logic calculations each genotype could perform. Such a measure of complexity is similar to a measure of phenotypic complexity used previously [5] in population genetics. The relative fitness was calculated by dividing the analyzed fitness value by the ancestor’s fitness (0.244898).

To examine why certain population sizes evolved larger genomes, we examined the “line of descent” (LOD) of the fittest type [65]. An LOD contains every intermediate genotype between the final individual with the greatest fitness and the ancestral genotype that initialized each population. This line provides a perfect “fossil record” to examine all of the mutations, insertions, and deletions that led to the final fittest genotype for each population. We also calculated the selection coefficient *s* for each mutation, defined as the ratio of the offspring’s fitness to the parent’s fitness minus one. We defined beneficial mutations as those with *s* > 0 and deleterious mutations as those with *s* < 0 (this ignores classifying slightly beneficial and slightly deleterious mutations as neutral.) We determined the number of beneficial insertion mutations by counting those insertions on the LOD with $s>\frac{1}{N}$, where *N* is the population size. These are beneficial mutations that are not nearly-neutral and hence should be under positive selection. We note that using $s>\frac{1}{N}$ is only an approximation, as the equation for a nearly neutral mutation is $\left|s\right|\ll \frac{1}{N_{e}}$, where *N*_*e*_ is the effective population size [77]. We also examined those mutations that had a slightly deleterious effect on fitness, i.e., those whose selection coefficient was $-\frac{1}{N}<s<0$.

## Supporting Information

S1 Table Merit rewards for the evolution of phenotypic traits. (PDF)

S1 Fig Evolution of complexity for small population sizes. Statistics shown in the main text for population sizes ranging from 10 to 100 individuals. Data for populations with 10 and 100 individuals are the same as in the main text. A: Evolution of genome size. B: Proportion of fixed insertions that were slightly-deleterious. C: Proportion of fixed insertions that were under positive selection. D: Number of evolved novel phenotypic traits. Red lines are the median values for each population size. The upper and lower limits of each box denote the third and first quartile, respectively. Whiskers are 1.5 times the relevant quartile value. Plus signs denote those data points beyond the whiskers. Data represent only those populations that did not go extinct.
(PDF)

S2 Fig The number of insertions fixed as a function of population size. Red lines are the median values for each population size. The upper and lower limits of each box denote the third and first quartile, respectively. Whiskers are 1.5 times the relevant quartile value. Plus signs denote those data points beyond the whiskers. Data represent only those populations that did not go extinct.
(PDF)

S3 Fig The evolved fitness relative to the ancestor genotype. Red lines are the median values for each population size. The upper and lower limits of each box denote the third and first quartile, respectively. Whiskers are 1.5 times the relevant quartile value. Plus signs denote those data points beyond the whiskers. Data represent only those populations that did not go extinct.
(PDF)

S4 Fig The effect of a fixed mutation rate on the evolution of phenotypic complexity. The variable genomic mutation rate treatment represents the data from when the genomic point mutation rate is 10^−2^ × *L*, were *L* is the genome size. The fixed genomic mutation rate treatment represents the data from when the genomic point mutation rate was fixed at 1.5 × 10^−1^, independent of the genome size. Red lines are the median values for each population size. The upper and lower limits of each box denote the third and first quartile, respectively. Whiskers are 1.5 times the relevant quartile value. Plus signs denote those data points beyond the whiskers. Data represent only those populations that did not go extinct.
(PDF)

S5 Fig The evolution of complexity in the non-functional insertion treatment. All subplots are a function of the population size. A: The final genome size. B: The final number of evolved phenotypic traits. Populations with 20 individuals are shown instead of those with ten individuals due to the high extinction rates of populations with ten individuals. Red lines are the median values for each population size. The upper and lower limits of each box denote the third and first quartile, respectively. Whiskers are 1.5 times the relevant quartile value. Plus signs denote those data points beyond the whiskers. Data represent only those populations that did not go extinct.
(PDF)

S6 Fig The proportion of fixed insertions that were under positive selection in the non-functional insertion treatment compared to the original treatment for populations with 10^4^ individuals. Red lines are the median values for each population size. The upper and lower limits of each box denote the third and first quartile, respectively. Whiskers are 1.5 times the relevant quartile value. Plus signs denote those data points beyond the whiskers. Data represent only those populations that did not go extinct.
(PDF)

S7 Fig The evolution of complexity in the deletion bias treatment. All subplots are a function of the population size. A: The final genome size. B: The final number of evolved phenotypic traits. Red lines are the median values for each population size. The upper and lower limits of each box denote the third and first quartile, respectively. Whiskers are 1.5 times the relevant quartile value. Plus signs denote those data points beyond the whiskers. Data represent only those populations that did not go extinct.
(PDF)

## References

[1] Bonner JT. The evolution of complexity by means of natural selection. Princeton: Princeton University Press; 1988.

[2] Adami C, Ofria C, Collier TC. Evolution of biological complexity. Proceedings of the National Academy of Sciences. 2000;97:4463–4468. 10.1073/pnas.97.9.4463 PMC18257 10781045

[3] Koonin EV. A non-adaptationist perspective on evolution of genomic complexity or the continued dethroning of man. Cell Cycle. 2004;3:278–283. 10.4161/cc.3.3.745 14726650

[4] Lynch M. The frailty of adaptive hypotheses for the origins of organismal complexity. Proceedings of the National Academy of Sciences. 2007;104(Suppl 1):8597–8604. 10.1073/pnas.0702207104 17494740 PMC1876435

[5] Tenaillon O, Silander OK, Uzan JP, Chao L. Quantifying organismal complexity using a population genetic approach. PLoS One. 2007;2:e217 10.1371/journal.pone.0000217 17299597 PMC1790863

[6] McShea DW, Brandon RN. Biology’s first law: the tendency for diversity and complexity to increase in evolutionary systems. Chicago: University of Chicago Press; 2010.

[7] Kimura M. The neutral theory of molecular evolution. New York: Cambridge University Press; 1984.

[8] Ohta T. The nearly neutral theory of molecular evolution. Annual Review of Ecology and Systematics. 1992;23:263–286. 10.1146/annurev.es.23.110192.001403

[9] Gillespie JH. Genetic drift in an infinite population: the pseudohitchhiking model. Genetics. 2000;155:909–919. 10835409 10.1093/genetics/155.2.909 PMC1461093

[10] Lynch M, Bobay LM, Catania F, Gout JF, Rho M. The repatterning of eukaryotic genomes by random genetic drift. Annual Review of Genomics and Human Genetics. 2011;12:347–366. 10.1146/annurev-genom-082410-101412 21756106 PMC4519033

[11] Eddy SR. The C-value paradox, junk DNA and ENCODE. Current Biology. 2012;22:R898–R899. 10.1016/j.cub.2012.10.002 23137679

[12] Palazzo AF, Gregory TR. The case for junk DNA. PLoS Genet. 2014;10:e1004351 10.1371/journal.pgen.1004351 24809441 PMC4014423

[13] Travisano M, Mongold JA, Bennett AF, Lenski RE. Experimental tests of the roles of adaptation, chance, and history in evolution. Science. 1995;267:87–90. 10.1126/science.7809610 7809610

[14] Wagenaar DA, Adami C. Influence of chance, history, and adaptation on digital evolution. Artificial Life. 2004;10:181–190. 10.1162/106454604773563603 15107230

[15] Lachapelle J, Reid J, Colegrave N. Repeatability of adaptation in experimental populations of different sizes. Proceedings of the Royal Society of London B: Biological Sciences. 2015;282:20143033 10.1098/rspb.2014.3033 25788593 PMC4389614

[16] Blount ZD, Borland CZ, Lenski RE. Historical contingency and the evolution of a key innovation in an experimental population of Escherichia coli. Proceedings of the National Academy of Sciences. 2008;105:7899–7906. 10.1073/pnas.0803151105 18524956 PMC2430337

[17] Meyer JR, Dobias DT, Weitz JS, Barrick JE, Quick RT, Lenski RE. Repeatability and contingency in the evolution of a key innovation in phage lambda. Science. 2012;335:428–432. 10.1126/science.1214449 22282803 PMC3306806

[18] Wagner A. The origins of evolutionary innovations: a theory of transformative change in living systems. New York: Oxford University Press; 2011 10.1093/acprof:oso/9780199692590.003.0095

[19] Kawecki TJ, Lenski RE, Ebert D, Hollis B, Olivieri I, Whitlock MC. Experimental evolution. Trends in Ecology & Evolution. 2012;27:547–560. 10.1016/j.tree.2012.06.001 22819306

[20] Masel J. Genetic drift. Current Biology. 2011;21:R837–R838. 10.1016/j.cub.2011.08.007 22032182

[21] Schoustra SE, Bataillon T, Gifford DR, Kassen R, et al The properties of adaptive walks in evolving populations of fungus. PLoS biology. 2009;7:2474 10.1371/journal.pbio.1000250 19956798 PMC2772970

[22] Quandt EM, Gollihar J, Blount ZD, Ellington AD, Georgiou G, Barrick JE. Fine-tuning citrate synthase flux potentiates and refines metabolic innovation in the Lenski evolution experiment. eLife. 2015;4:e09696 10.7554/eLife.09696 26465114 PMC4718724

[23] Burmeister A, Lenski R, Meyer J. Host coevolution alters the adaptive landscape of a virus; 2016. Proceedings of the Royal Society of London B: Biological Sciences. 2016;283:20161528 10.1098/rspb.2016.1528 27683370 PMC5046904

[24] Wagner A. Neutralism and selectionism: a network-based reconciliation. Nature Reviews Genetics. 2008;9:965–974. 10.1038/nrg2473 18957969

[25] Wright S. The roles of mutation, inbreeding, crossbreeding, and selection in evolution. In: Proceedings of the Sixth International Congress of Genetics. vol. 1; 1932. p. 356–366.

[26] Rozen DE, Habets MGJL, Handel A, De Visser JAGM. Heterogeneous adaptive trajectories of small populations on complex fitness landscapes. PLoS One. 2008;3:e1715–e1715. 10.1371/journal.pone.0001715 18320036 PMC2248617

[27] Jain K, Krug J, Park SC. Evolutionary advantage of small populations on complex fitness landscapes. Evolution. 2011;65:1945–1955. 10.1111/j.1558-5646.2011.01280.x 21729050

[28] Lynch M, Conery JS. The origins of genome complexity. Science. 2003;302:1401–1404. 10.1126/science.1089370 14631042

[29] Lynch M. The origins of genome architecture. Sunderland: Sinauer Associates; 2007.

[30] Lynch M. Streamlining and simplification of microbial genome architecture. Annu Rev Microbiol. 2006;60:327–349. 10.1146/annurev.micro.60.080805.142300 16824010

[31] Batut B, Knibbe C, Marais G, Daubin V. Reductive genome evolution at both ends of the bacterial population size spectrum. Nature Reviews Microbiology. 2014;12:841–850. 10.1038/nrmicro3331 25220308

[32] Zwart MP, Willemsen A, Daròs JA, Elena SF. Experimental evolution of pseudogenization and gene loss in a plant RNA virus. Molecular Biology and Evolution. 2014;31:121–134. 10.1093/molbev/mst175 24109604 PMC3879446

[33] Koonin EV. The logic of chance: the nature and origin of biological evolution. Upper Saddle River: FT press; 2012.

[34] Weissman DB, Desai MM, Fisher DS, Feldman MW. The rate at which asexual populations cross fitness valleys. Theoretical Population Biology. 2009;75:286–300. 10.1016/j.tpb.2009.02.006 19285994 PMC2992471

[35] Weissman DB, Feldman MW, Fisher DS. The rate of fitness-valley crossing in sexual populations. Genetics. 2010;186:1389–1410. 10.1534/genetics.110.123240 20923976 PMC2998319

[36] Ochs IE, Desai MM. The competition between simple and complex evolutionary trajectories in asexual populations. BMC Evolutionary Biology. 2015;15:55 10.1186/s12862-015-0334-0 25881244 PMC4391547

[37] Adami C. Digital genetics: Unravelling the genetic basis of evolution. Nature Reviews Genetics. 2006;7:109–118. 10.1038/nrg1771 16418746

[38] Yedid G, Bell G. Macroevolution simulated with autonomously replicating computer programs. Nature. 2002;420:810–812. 10.1038/nature01151 12490948

[39] Bell G; The Royal Society. Experimental macroevolution. Proc R Soc B. 2016;283:20152547. 10.1098/rspb.2015.2547 26763705 PMC4721102

[40] Batut B, Parsons DP, Fischer S, Beslon G, Knibbe C. In silico experimental evolution: A tool to test evolutionary scenarios. BMC Bioinformatics. 2013;14:1 10.1186/1471-2105-14-S15-S11 24564457 PMC3851946

[41] Elena SF, Lenski RE. Evolution experiments with microorganisms: The dynamics and genetic bases of adaptation. Nature Reviews Genetics. 2003;4:457–469. 10.1038/nrg1088 12776215

[42] Ofria C, Bryson DM, Wilke CO. Avida: A Software Platform for Research in Computational Evolutionary Biology In: Komosinski Maciej, A A, editor. Artificial Life Models in Software. Springer London; 2009 p. 3–35. 10.1007/978-1-84882-285-6_1 15107231

[43] Adami C. Introduction to Artificial Life. New York: Springer Verlag; 1998.

[44] Wilke CO, Adami C. The biology of digital organisms. Trends in Ecology & Evolution. 2002;17:528–532. 10.1016/S0169-5347(02)02612-5

[45] Mira A, Ochman H, Moran NA. Deletional bias and the evolution of bacterial genomes. Trends in Genetics. 2001;17:589–596. 10.1016/S0168-9525(01)02447-7 11585665

[46] Kuo CH, Ochman H. Deletional bias across the three domains of life. Genome Biology and Evolution. 2009;1:145–152. 10.1093/gbe/evp016 20333185 PMC2817411

[47] Vinogradov AE. Selfish DNA is maladaptive: Evidence from the plant Red List. Trends in Genetics. 2003;19:609–614. 10.1016/j.tig.2003.09.010 14585612

[48] Vinogradov AE. Genome size and extinction risk in vertebrates. Proceedings of the Royal Society of London B: Biological Sciences. 2004;271:1701–1706. 10.1098/rspb.2004.2776 15306290 PMC1691778

[49] Muller HJ. The relation of recombination to mutational advance. Mutation Research. 1964;1:2–9. 10.1016/0027-5107(64)90047-8 14195748

[50] Lynch M, Bürger R, Butcher D, Gabriel W. The mutational meltdown in asexual populations. Journal of Heredity. 1993;84:339–344. 8409355 10.1093/oxfordjournals.jhered.a111354

[51] Zeyl C, Mizesko M, De Visser J. Mutational meltdown in laboratory yeast populations. Evolution. 2001;55:909–917. 10.1111/j.0014-3820.2001.tb00608.x 11430651

[52] Maynard Smith J. The causes of extinction. Philosophical Transactions of the Royal Society B. 1989;325:241–252. 10.1098/rstb.1989.0086 2574882

[53] Willi Y, Van Buskirk J, Hoffmann AA. Limits to the adaptive potential of small populations. Annual Review of Ecology, Evolution, and Systematics. 2006;37:433–458. 10.1146/annurev.ecolsys.37.091305.110145

[54] Lynch M, Conery JS. The evolutionary fate and consequences of duplicate genes. Science. 2000;290:1151–1155. 10.1126/science.290.5494.1151 11073452

[55] Hughes AL. The evolution of functionally novel proteins after gene duplication. Proceedings of the Royal Society of London B. 1994;256:119–124. 10.1098/rspb.1994.0058 8029240

[56] Bergthorsson U, Andersson DI, Roth JR. Ohno’s dilemma: Evolution of new genes under continuous selection. Proceedings of the National Academy of Sciences. 2007;104:17004–17009. 10.1073/pnas.0707158104 17942681 PMC2040452

[57] Blount ZD, Barrick JE, Davidson CJ, Lenski RE. Genomic analysis of a key innovation in an experimental Escherichia coli population. Nature. 2012;489:513–518. 10.1038/nature11514 22992527 PMC3461117

[58] Näsvall J, Sun L, Roth JR, Andersson DI. Real-time evolution of new genes by innovation, amplification, and divergence. Science. 2012;338:384–387. 10.1126/science.1226521 23087246 PMC4392837

[59] Walsh JB. How often do duplicated genes evolve new functions? Genetics. 1995;139:421–428. 7705642 10.1093/genetics/139.1.421 PMC1206338

[60] Daubin V, Moran NA. Comment on “The origins of genome complexity”. Science. 2004;306:978–978. 10.1126/science.1100559 15528429

[61] Kuo CH, Moran NA, Ochman H. The consequences of genetic drift for bacterial genome complexity. Genome Research. 2009;19:1450–1454. 10.1101/gr.091785.109 19502381 PMC2720180

[62] Weinreich DM, Delaney NF, DePristo MA, Hartl DL. Darwinian evolution can follow only very few mutational paths to fitter proteins. Science. 2006;312:111–114. 10.1126/science.1123539 16601193

[63] Palmer AC, Toprak E, Baym M, Kim S, Veres A, Bershtein S, et al Delayed commitment to evolutionary fate in antibiotic resistance fitness landscapes. Nature Communications. 2015;6:7385 10.1038/ncomms8385 26060115 PMC4548896

[64] Elena SF, Ekunwe L, Hajela N, Oden SA and Lenski RE. Distribution of fitness effects caused by random insertion mutations in *Escherichia coli*. Genetica. 1988;102–103:349–358. 10.1007/978-94-011-5210-5_28 9720287

[65] Lenski RE, Ofria C, Pennock RT, Adami C. The evolutionary origin of complex features. Nature. 2003;423:139–144. 10.1038/nature01568 12736677

[66] Elena SF, Wilke CO, Ofria C, Lenski RE. Effects of population size and mutation rate on the evolution of mutational robustness. Evolution. 2007;61:666–674. 10.1111/j.1558-5646.2007.00064.x 17348929

[67] Otto SP. The evolutionary enigma of sex. The American Naturalist. 2009;174:S1–S14. 10.1086/599084 19441962

[68] Lynch M, Conery J, Burger R. Mutational meltdowns in sexual populations. Evolution. 1995;49:1067–1080. 10.2307/2410432 28568521

[69] Misevic D, Ofria C, Lenski RE. Sexual reproduction reshapes the genetic architecture of digital organisms. Proceedings of the Royal Society of London B. 2006;273:457–464. 10.1098/rspb.2005.3338 16615213 PMC1560214

[70] Walker BL, Ofria C. Evolutionary potential is maximized at intermediate diversity levels. In: Adami C, Bryson DM, Ofria C, Pennock R, editors. Proceedings of the 13th International Conference on the Synthesis and Simulation of Living Systems. Cambridge, MA: MIT Press; 2012. p. 116–120.

[71] Zaman L, Meyer JR, Devangam S, Bryson DM, Lenski RE, Ofria C. Coevolution drives the emergence of complex traits and promotes evolvability. PLoS Biology. 2014;12:e1002023 10.1371/journal.pbio.1002023 25514332 PMC4267771

[72] Pennock RT. Models, simulations, instantiations, and evidence: the case of digital evolution. Journal of Experimental & Theoretical Artificial Intelligence. 2007;19:29–42. 10.1080/09528130601116113

[73] Hindré T, Knibbe C, Beslon G, Schneider D. New insights into bacterial adaptation through in vivo and in silico experimental evolution. Nature Reviews Microbiology. 2012;10:352–365. 10.1038/nrmicro2750 22450379

[74] Lenski RE, Ofria C, Collier TC, Adami C. Genome complexity, robustness and genetic interactions in digital organisms. Nature. 1999;400:661–664. 10.1038/23245 10458160

[75] Goldsby HJ, Knoester DB, Ofria C, Kerr B. The evolutionary origin of somatic cells under the dirty work hypothesis. PLoS Biology. 2014;12:e1001858 10.1371/journal.pbio.1001858 24823361 PMC4019463

[76] Lenski RE, Rose MR, Simpson SC, Tadler SC. Long-term experimental evolution in Escherichia coli. I. Adaptation and divergence during 2,000 generations. The American Naturalist. 1991;138:1315–1341. 10.1086/285289

[77] Ohta T, Gillespie JH. Development of neutral and nearly neutral theories. Theoretical population biology. 1996;49:128–142. 10.1006/tpbi.1996.0007 8813019

