# Supplementary material for: Different Evolutionary Paths to Complexity for Small and Large Populations of Digital Organisms
Source: PLoS Comput Biol. 2016 Dec 6;12(12):e1005066. doi: 10.1371/journal.pcbi.1005066 (PMC5140054; doi:10.1371/journal.pcbi.1005066)
Supplement: S1 Table — (PDF) [file pcbi.1005066.s001.pdf]

| Boolean Logic Calculaton | Merit Multiplier |
|---|---|
| NOT | 2 |
| NAND | 2 |
| ORNOT | 4 |
| AND | 4 |
| ANDNOT | 8 |
| OR | 8 |
| NOR | 16 |
| XOR | 16 |
| XNOR (Equals) | 32 |
