# Supplementary figures and images for: Different Evolutionary Paths to Complexity for Small and Large Populations of Digital Organisms
Source: PLoS Comput Biol. 2016 Dec 6;12(12):e1005066. doi: 10.1371/journal.pcbi.1005066 (PMC5140054; doi:10.1371/journal.pcbi.1005066)

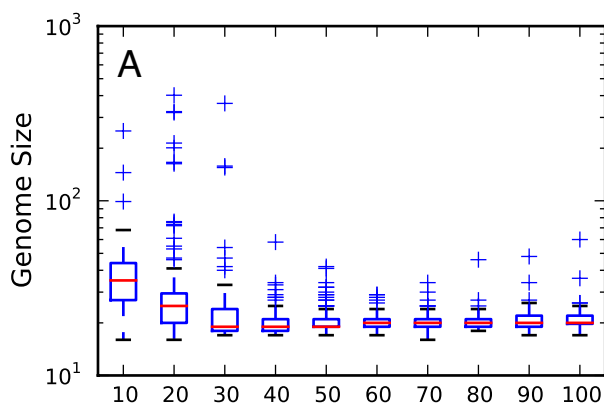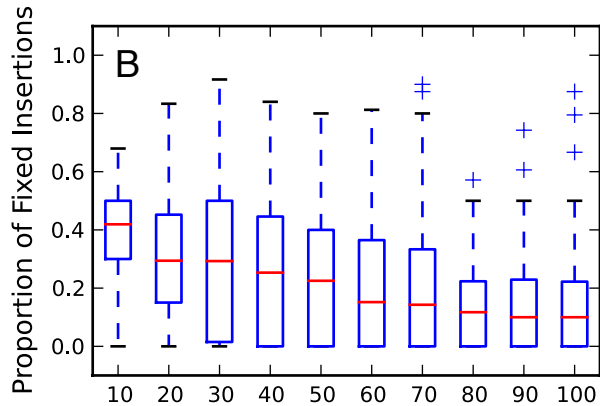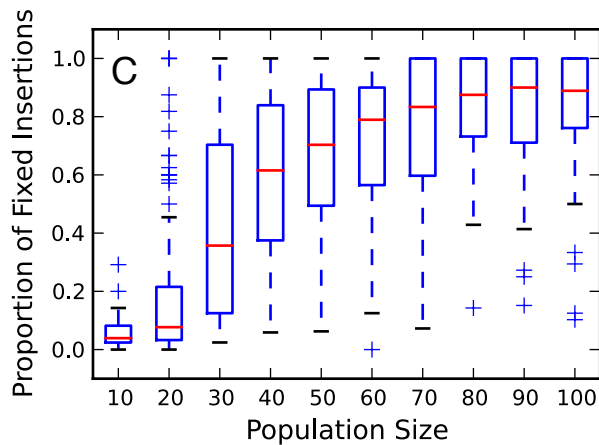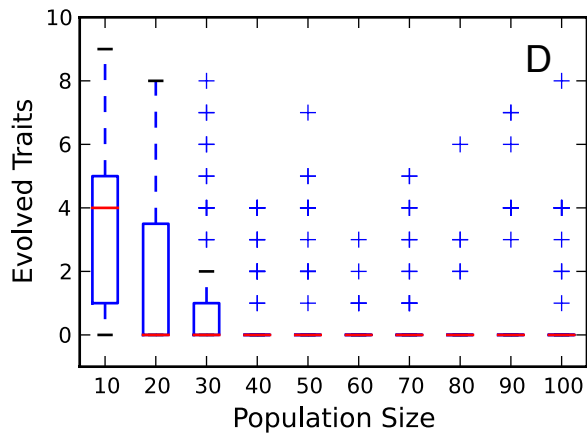

Supplement: S1 Fig — Statistics shown in the main text for population sizes ranging from 10 to 100 individuals. Data for populations with 10 and 100 individuals are the same as in the main text. A: Evolution of genome size. B: Proportion of fixed insertions that were slightly-deleterious. C: Proportion of fixed insertions that were under positive selection. D: Number of evolved novel phenotypic traits. Red lines are the median values for each population size. The upper and lower limits of each box denote the third and first quartile, respectively. Whiskers are 1.5 times the relevant quartile value. Plus signs denote those data points beyond the whiskers. Data represent only those populations that did not go extinct. (PDF) [file pcbi.1005066.s002.pdf]

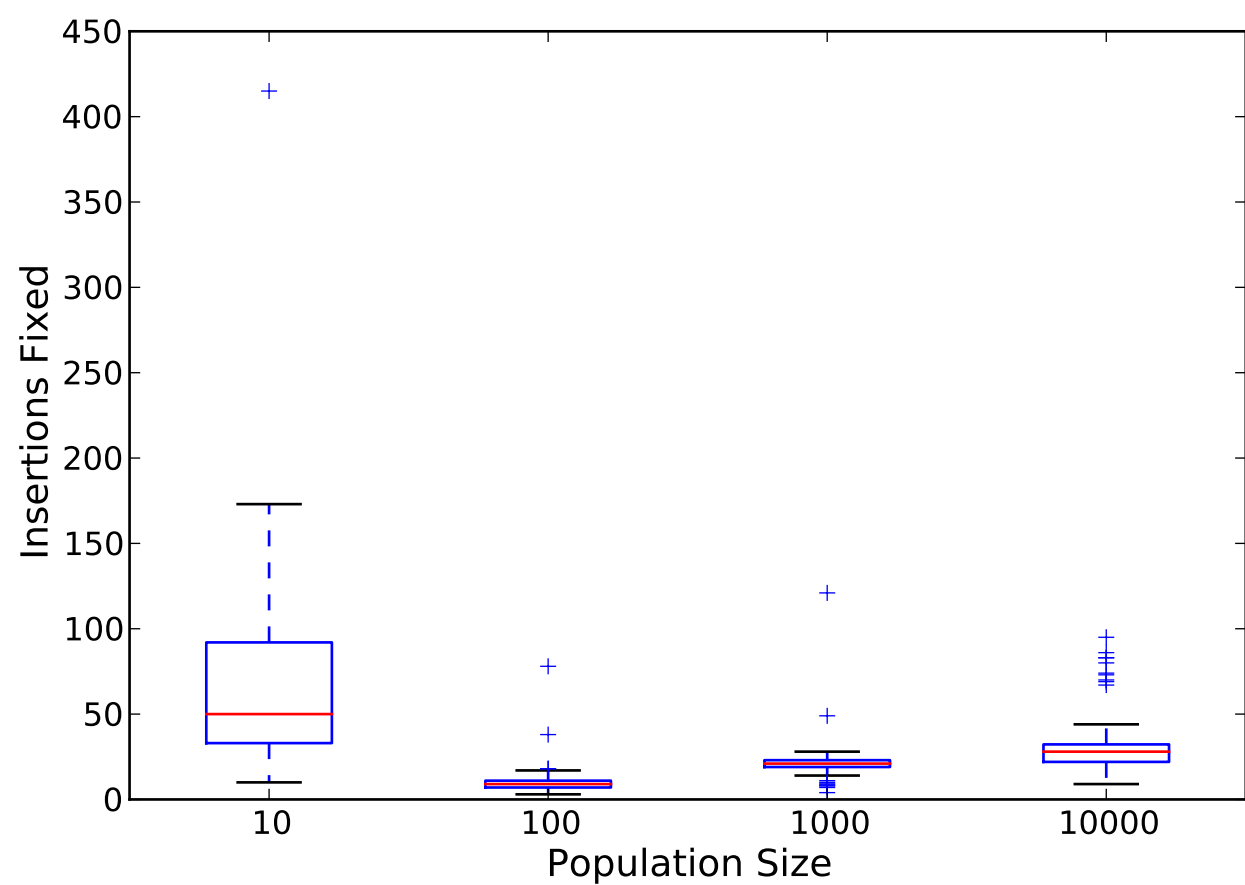

Supplement: S2 Fig — Red lines are the median values for each population size. The upper and lower limits of each box denote the third and first quartile, respectively. Whiskers are 1.5 times the relevant quartile value. Plus signs denote those data points beyond the whiskers. Data represent only those populations that did not go extinct. (PDF) [file pcbi.1005066.s003.pdf]

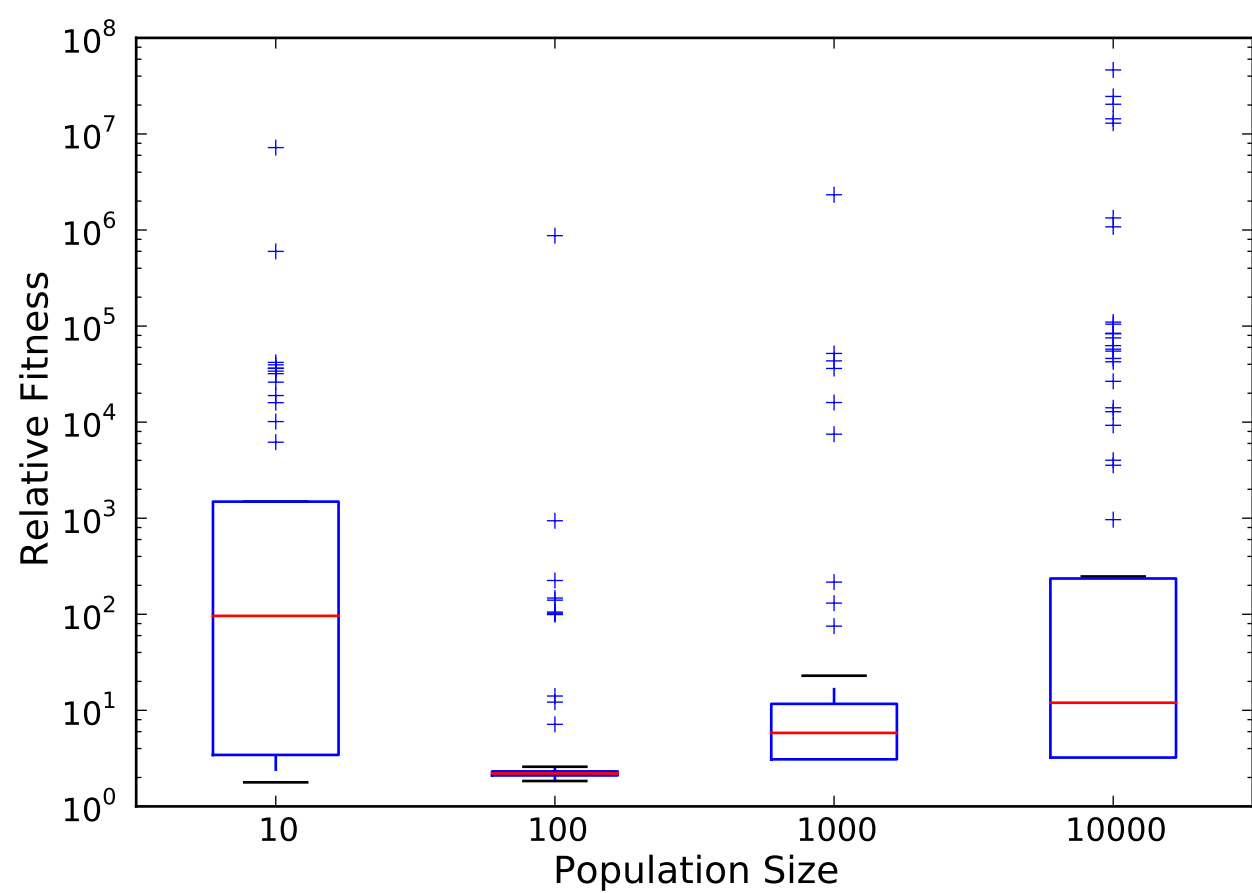

Supplement: S3 Fig — Red lines are the median values for each population size. The upper and lower limits of each box denote the third and first quartile, respectively. Whiskers are 1.5 times the relevant quartile value. Plus signs denote those data points beyond the whiskers. Data represent only those populations that did not go extinct. (PDF) [file pcbi.1005066.s004.pdf]

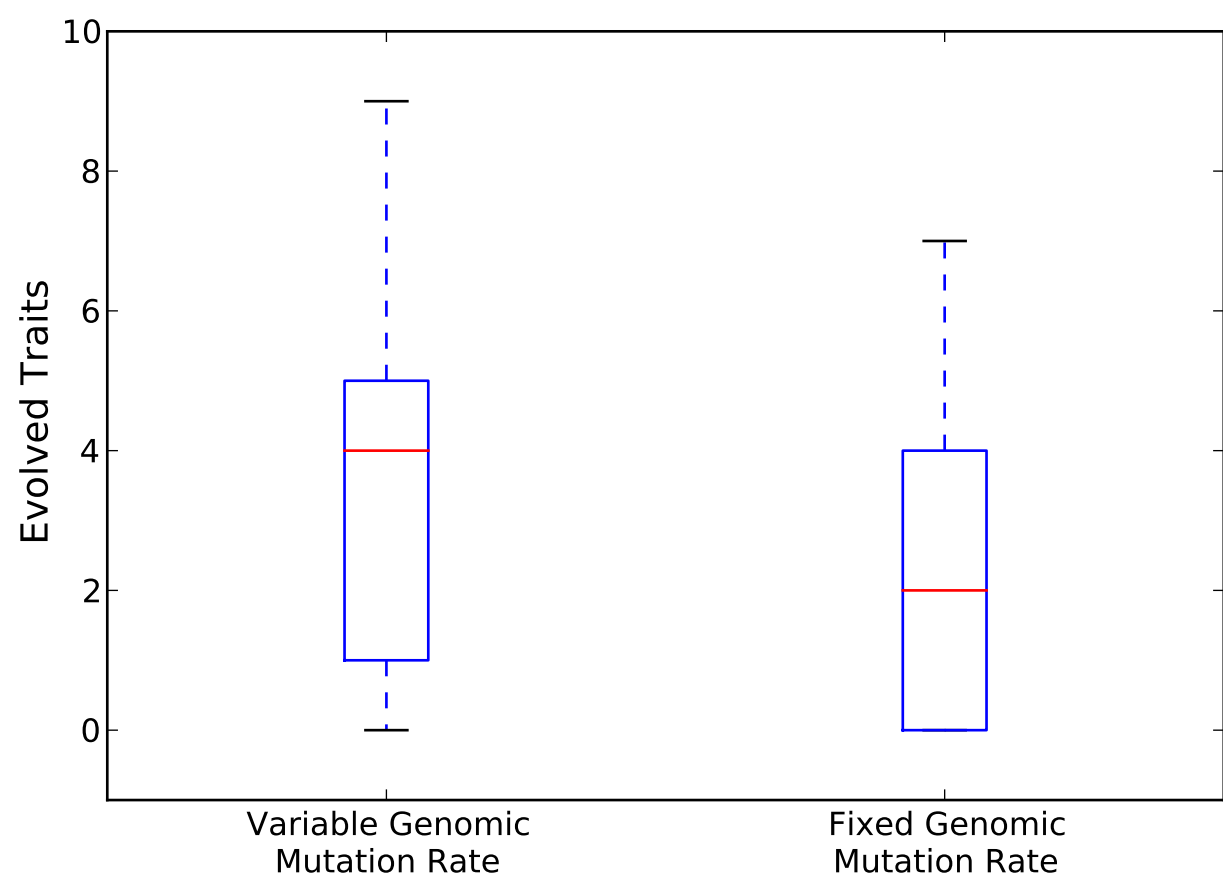

Supplement: S4 Fig — The variable genomic mutation rate treatment represents the data from when the genomic point mutation rate is 10−2 × L, were L is the genome size. The fixed genomic mutation rate treatment represents the data from when the genomic point mutation rate was fixed at 1.5 × 10^−1^, independent of the genome size. Red lines are the median values for each population size. The upper and lower limits of each box denote the third and first quartile, respectively. Whiskers are 1.5 times the relevant quartile value. Plus signs denote those data points beyond the whiskers. Data represent only those populations that did not go extinct. (PDF) [file pcbi.1005066.s005.pdf]

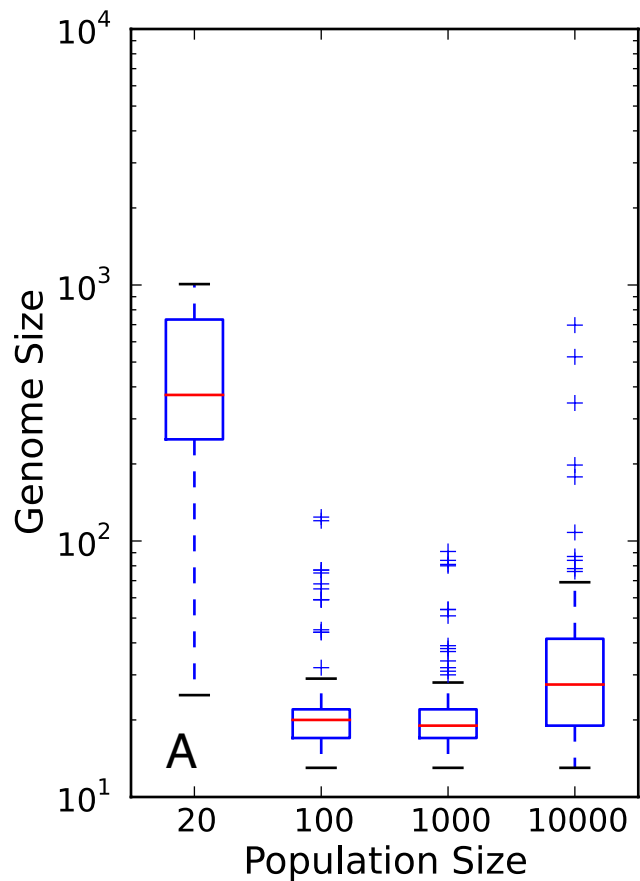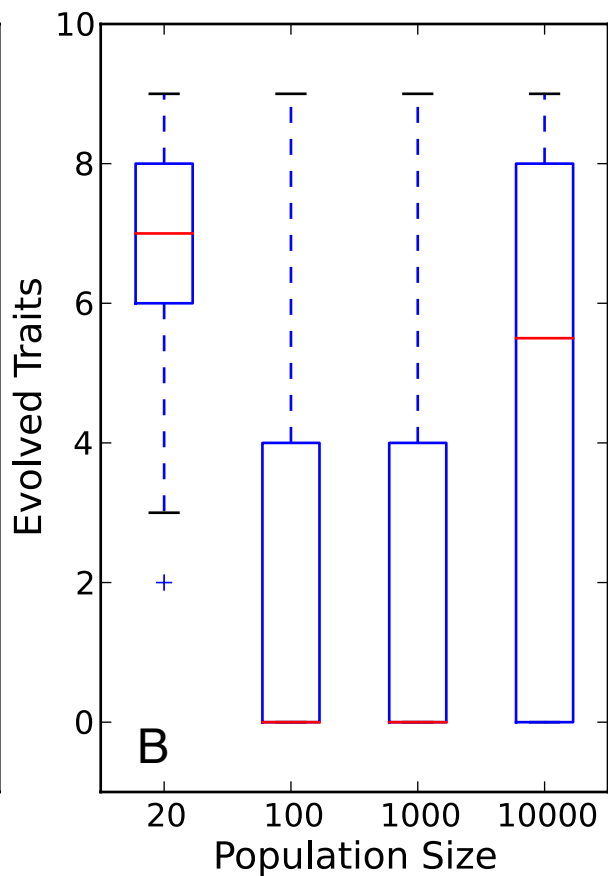

Supplement: S5 Fig — All subplots are a function of the population size. A: The final genome size. B: The final number of evolved phenotypic traits. Populations with 20 individuals are shown instead of those with ten individuals due to the high extinction rates of populations with ten individuals. Red lines are the median values for each population size. The upper and lower limits of each box denote the third and first quartile, respectively. Whiskers are 1.5 times the relevant quartile value. Plus signs denote those data points beyond the whiskers. Data represent only those populations that did not go extinct. (PDF) [file pcbi.1005066.s006.pdf]

Proportion of Fixed Insertions

1.0  
0.8  
0.6  
0.4  
0.2  
0.0

Original  
Treatment

Non-Functional Insertion  
Treatment

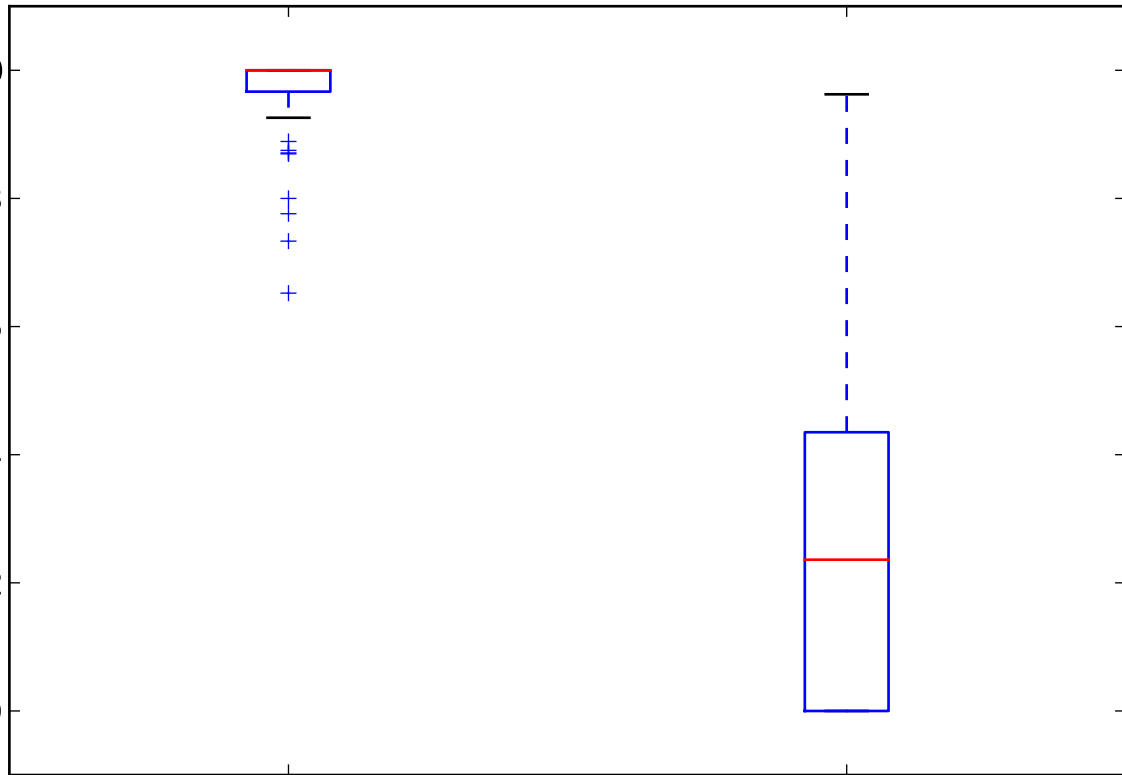

Supplement: S6 Fig — Red lines are the median values for each population size. The upper and lower limits of each box denote the third and first quartile, respectively. Whiskers are 1.5 times the relevant quartile value. Plus signs denote those data points beyond the whiskers. Data represent only those populations that did not go extinct. (PDF) [file pcbi.1005066.s007.pdf]

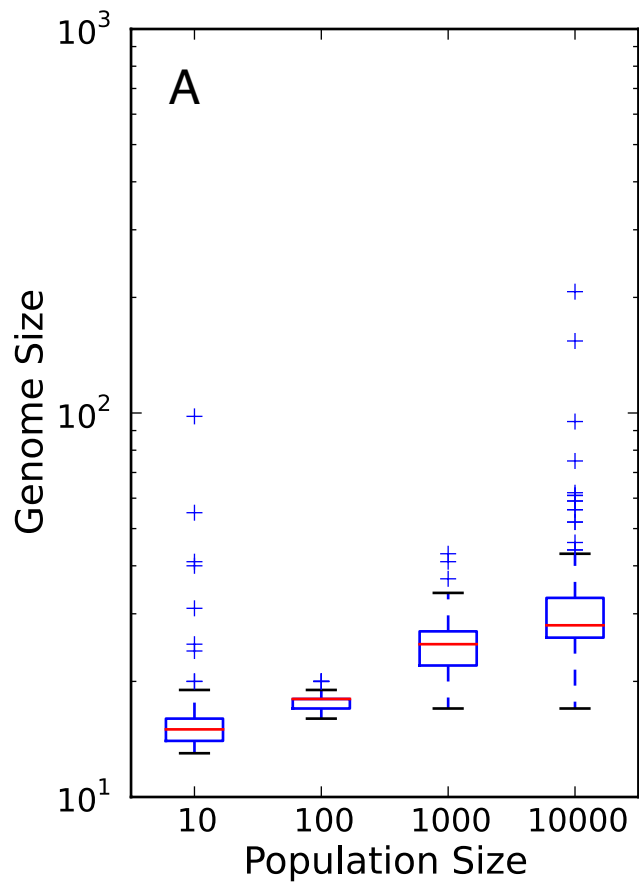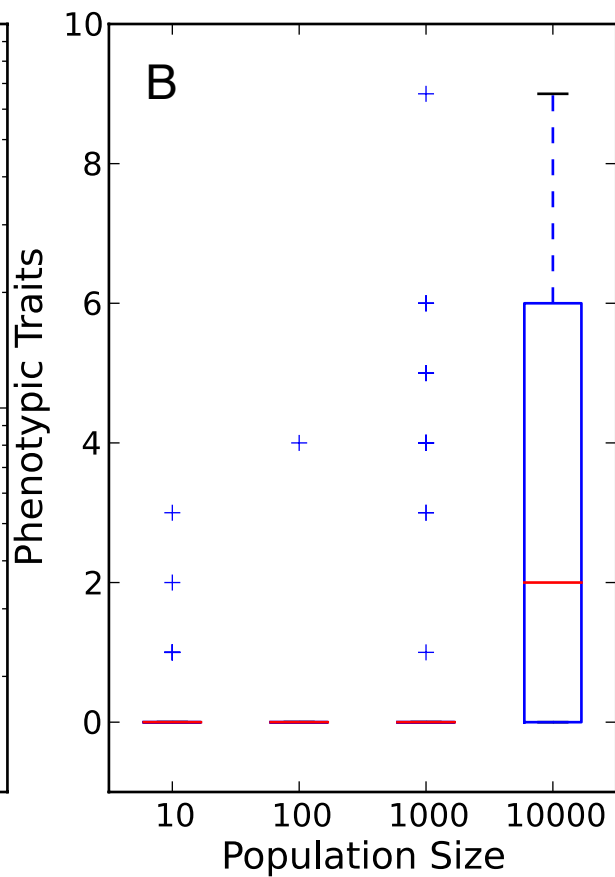

Supplement: S7 Fig — All subplots are a function of the population size. A: The final genome size. B: The final number of evolved phenotypic traits. Red lines are the median values for each population size. The upper and lower limits of each box denote the third and first quartile, respectively. Whiskers are 1.5 times the relevant quartile value. Plus signs denote those data points beyond the whiskers. Data represent only those populations that did not go extinct. (PDF) [file pcbi.1005066.s008.pdf]
